# Lung Ultrasound Score in COVID-19 Patients Correlates with PO_2_/FiO_2_, Intubation Rates, and Mortality

**DOI:** 10.5811/westjem.59975

**Published:** 2023-12-22

**Authors:** Shin-Yi Lai, Jesse M Schafer, Mary Meinke, Tyler Beals, Michael Doff, Anne Grossestreuer, Beatrice Hoffmann

**Affiliations:** * Beth Israel Deaconess Medical Center, Department of Emergency Medicine, Boston, Massachusetts; † St Vincent Hospital, Department of Emergency Medicine, Associated Physicians of Harvard Medical Faculty Physicians, Worcester, Massachusetts

## Abstract

**Introduction:**

The point-of-care lung ultrasound (LUS) score has been used in coronavirus 2019 (COVID-19) patients for diagnosis and risk stratification, due to excellent sensitivity and infection control concerns. We studied the ratio of partial pressure of oxygen in arterial blood to the fraction of inspiratory oxygen concentration (PO_2_/FiO_2_), intubation rates, and mortality correlation to the LUS score.

**Methods:**

We conducted a systematic review using PRISMA guidelines. Included were articles published from December 1, 2019–November 30, 2021 using LUS in adult COVID-19 patients in the intensive care unit or the emergency department. Excluded were studies on animals and on pediatric and pregnant patients. We assessed bias using QUADAS-2. Outcomes were LUS score and correlation to PO2/FiO2, intubation, and mortality rates. Random effects model pooled the meta-analysis results.

**Results:**

We reviewed 27 of 5,267 studies identified. Of the 27 studies, seven were included in the intubation outcome, six in the correlation to PO_2_/FiO_2_ outcome, and six in the mortality outcome. Heterogeneity was found in ultrasound protocols and outcomes. In the pooled results of 267 patients, LUS score was found to have a strong negative correlation to PO_2_/FiO_2_ with a correlation coefficient of −0.69 (95% confidence interval [CI] −0.75, −0.62). In pooled results, 273 intubated patients had a mean LUS score that was 6.95 points higher (95% CI 4.58–9.31) than that of 379 non-intubated patients. In the mortality outcome, 385 survivors had a mean LUS score that was 4.61 points lower (95% CI 3.64–5.58) than that of 181 non-survivors. There was significant heterogeneity between the studies as measured by the I^2^ and Cochran Q test.

**Conclusion:**

A higher LUS score was strongly correlated with a decreasing PO_2_/FiO_2_ in COVID-19 pneumonia patients. The LUS score was significantly higher in intubated vs non-intubated patients with COVID-19. The LUS score was significantly lower in critically ill patients with COVID-19 pneumonia that survive.

## INTRODUCTION

The novel severe acute respiratory syndrome coronavirus 2 (SARS-CoV-2), first described in December 2019,^1^ is responsible for an estimated 768 million infections and nearly 7 million deaths worldwide.^2^ Approximately 17–35% of hospitalized patients with coronavirus disease 2019 (COVID-19) develop hypoxemic respiratory failure and acute respiratory distress syndrome (ARDS) requiring intensive care unit (ICU) admission^2^ with invasive ventilation required in 29–91%.^3^ This wide variability reflects the evolution of pharmacotherapies and various practice patterns through different waves of the pandemic in addition to social and economic factors such as vaccination rates and availability of ICU-level resources in different countries.^4^ Given the scale of the pandemic and significant morbidity/mortality related to COVID-19, efforts have been undertaken toward the testing and identification of COVID-19 positive patients at risk for significant morbidity/mortality based on clinical or radiographic parameters.

Radiographic modalities commonly used in the evaluation of COVID-19 pneumonia lung involvement include chest radiograph (CXR) as well as computed tomography (CT). However, CXR may miss up to 45% of COVID-19 polymerase chain reaction (PCR)-confirmed cases^5^ ^,^ ^6^ and correlates poorly with the clinical picture compared to lung ultrasound (LUS) and CT.^6^ ^,^ ^7^ Computed tomography is considered the gold standard imaging modality for the investigation of patients with COVID-19 pneumonia^8^ but is limited by resource allocation and transport risks.^9^ ^,^ ^10^ Studies have found the sensitivity of LUS for COVID-19 diagnosis to be close to 86–90%^11^ ^,^ ^12^ when performed by experienced operators, with a 85–92% specificity,^13^ ^–^ ^15^ which is comparable to CT and PCR testing. Lung ultrasound has the added benefits of being inexpensive, noninvasive, free of radiation exposure, and easily repeated.

Due to workflow availability and infection control measures, bedside point-of-care ultrasound (POCUS) has increasingly been used in the diagnosis and risk stratification of emergency department (ED) patients as well as to monitor the progression of COVID-19 disease in the ICU.^16^ Ultrasound as a point-of-care imaging modality is well-suited to COVID-19 patients because COVID-19 lung changes are sonographically detectable and are prominent in the lung periphery.^17^ In particular, sonographic features of COVID-19 pneumonitis include increased number of B-lines, pleural line irregularities, and sub-pleural consolidations.^18^

The LUS score was introduced to grade ultrasound findings based on examination of several lung regions in the anterior, lateral, and posterior aspects of the left and right chest wall. Several protocols have been published and differ in the number of lung zones examined.^19^ ^–^ ^21^ Each region is scored according to four ultrasound aeration patterns with the final LUS score comprised of the sum of scores in the evaluated regions. Scores can range from 0-36 depending on the protocol and number of total examined lung fields. (See further illustration and detailed discussion of various LUS protocols by Allinovi et al in Supplement 1).^22^ A higher LUS score correlates with an increasing degree of pulmonary involvement^19^ and has been shown to correlate with disease severity and predicts mortality as highlighted by the Berlin criteria in patients with ARDS.^23^ ^,^ ^24^

Little is known about the correlation between LUS findings and abnormalities of gas exchange in COVID-19. The PO_2_/FiO_2_ ratio is considered a global index of tissue aeration.^25^ It is currently used to assess the severity of respiratory failure in patients with ARDS^26^ and correlates to mortality rate.^27^ In COVID-19, many patients present with respiratory alkalosis with hypoxia that does not correlate with pulse oximetry measurements.^28^ This is primarily due to the left shift of the oxygen–hemoglobin dissociation curve secondary to alkalosis and low pCO_2_ levels.^28^ Therefore, the PO_2_/FiO_2_ ratio is the standard measurement used for evaluation of blood oxygenation in these patients and was chosen as an outcome for analysis. The LUS score likely identifies the degree of damaged lung regions that contribute to hypoxemia through impaired aeration, vasoconstriction, and shunt,^29^ and it has a strong negative correlation with PO_2_/FiO_2_ values.

Our study objective was to determine whether the LUS score correlated with the clinical parameters of PO_2_/FiO_2_, intubation rates, and mortality, thus identifying patients at a high risk of clinical deterioration.

## METHODS

In accordance with systematic review guidelines, the study protocol was registered with the International Prospective Register of Systematic Reviews (PROSPERO ID CRD42020217983). We conducted a systematic review of the literature with principles from the Preferred Reporting Items for Systematic Review and Meta-analysis Protocols (PRISMA-P).^30^ ^,^ ^31^ Included studies evaluated patients ≥18 years of age who tested COVID-19 positive by confirmed PCR testing and used bedside LUS with a reported LUS numerical scoring system in the ED or ICU. We excluded animal studies, as well as studies on pediatric patients, asymptomatic patients, pregnant patients, those without PCR confirmation of COVID-19 pneumonia, and studies without a clear description of LUS abnormalities in numerical scoring. Outcome measures were intubation rates, mortality, and PO_2_/FiO_2_ ratio.

A comprehensive search for available research was performed by a health sciences librarian (MM) with expertise in systematic review search strategies. Databases Medline, Embase, Pubmed, Web of Science, Cochrane databases that mentioned POCUS, ultrasound and COVID-19, SARS CoV2, and LUS were searched until a cutoff date of November 30, 2021. The PROSPERO database was also queried for ongoing or recently completed systematic reviews. (The PUBMED search strategy is illustrated in Appendix 1.) Eligible studies selected for further assessment included the following: randomized and non-randomized controlled studies; prospective and retrospective cohort studies; and observational studies. We excluded case reports, non-original research, and letters to the editor.

**Figure 1.**
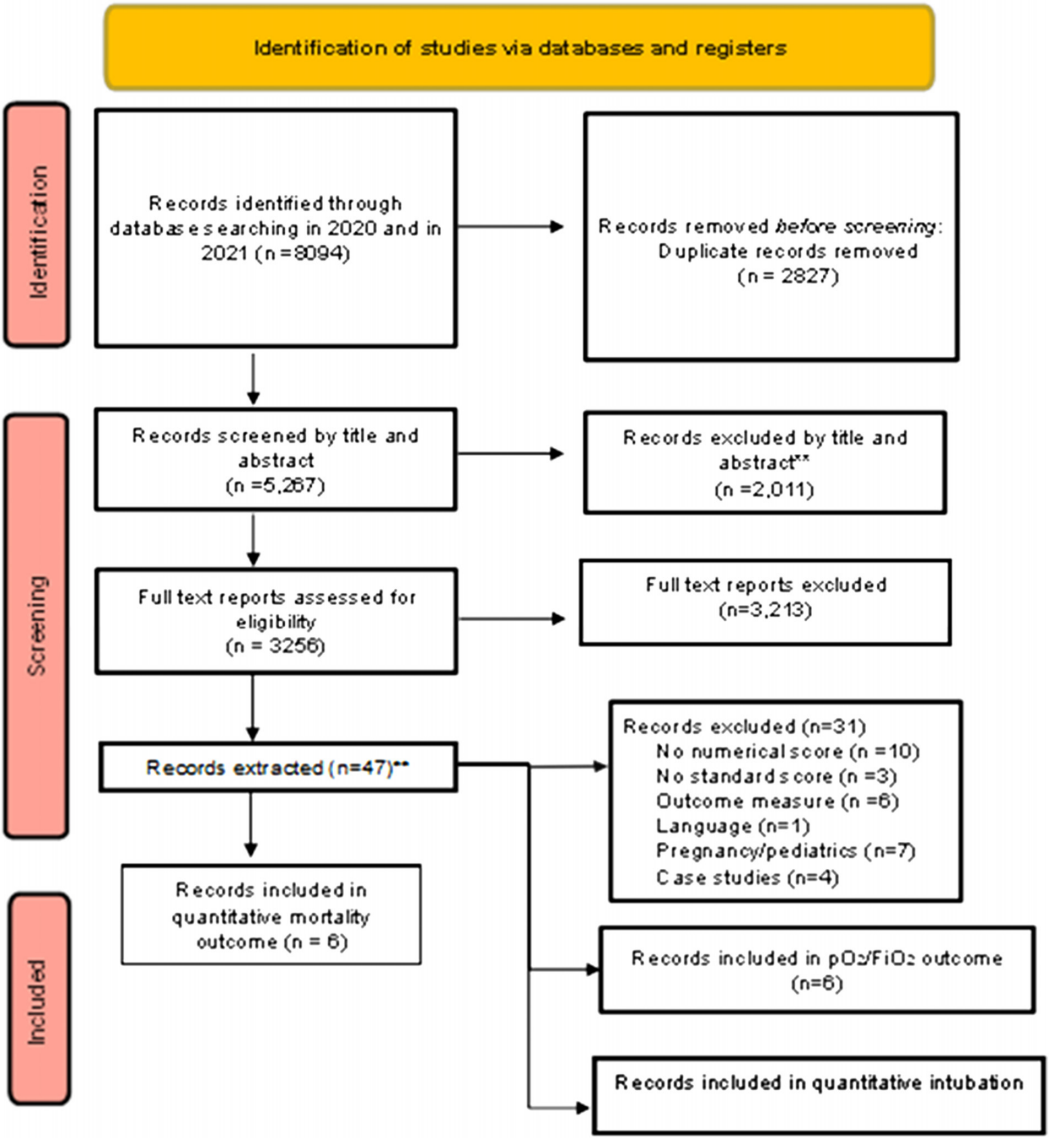
Preferred Reporting Items for Systematic Reviews and Meta-analysis extension for scoping review flow diagram (PRISMA-ScR). **Note: Studies included in meta-analysis (n = 16). Three articles are in more than one group: Bosso is in both mortality and PO_2_/FiO_2_ outcome; Rojatti is in both mortality and PO_2_/FiO_2_ outcome; and de Alencar is in both intubation and death outcome. *PO*_*2*_ */FiO*_*2*_, ratio of partial pressure of oxygen in arterial blood to the fraction of inspiratory oxygen concentration.

Search results were collected in EndNote X9. Two review authors individually screened the titles and abstracts yielded by the search against inclusion criteria. Review authors obtained full-text reports of titles that met inclusion criteria or where there was any uncertainty. The full-text reports were screened including whether they met including criteria. Disagreements were resolved through discussion and. if necessary, a third reviewer. A list of excluded studies was recorded based on the reasons for exclusion (Supplement 2). Results of the search and selection process are illustrated in Figure 1 and reported according to the PRISMA extension for scoping review flow diagram (PRISMA-ScR).^32^ The two initial review authors were not blinded to the journal titles, study authors, or institutions.

One reviewer extracted data for studies that met inclusion criteria by standardized forms. Extracted results were reviewed by a separate author to minimize errors. Data abstracted included study characteristics (author, journal reference, study design, inclusion/exclusion criteria, index text used, reference test used, general setting), demographic information, sample size, intervention details, experience of the operator, timing of the LUS protocol, and reported patient outcomes. Quantitative data on relative risk, odds ratio was extracted from original articles and collected using an Excel-based form (Microsoft Corp, Redmond, WA). We performed a meta-analysis in Cochrane RevMan 5.4 using a random effects model.^33^ For studies with missing outcomes, the original researchers were contacted for additional information.

We assessed the methodological quality of reported research using the QUADAS-2 tool (Bristol Medical School: Population Health Sciences, University of Bristol, UK).^34^ The domains were evaluated for each included study and are reported in Supplement 3. QUADAS-2 includes four main domains: patient selection; index test; reference standard; and flow and timing. In domain one, patient selection, we omitted the question “Was a case-control design avoided?” since we did not include any case series or case reports. In domain three, reference test, we added signal questions referring to operators’ expertise and background, technical features of the US hardware and appropriateness of the ultrasound protocol.

To reduce bias, the core outcome set was searched in COMET (Core Outcome Measures in Effectiveness Trials) Database.^35^ The Core Outcome Set for Clinical Trials on Coronavirus Disease 2019 (COS-COVID) had several outcomes for severity type (composite events, length of hospital stay, PaO_2_/FiO_2_, duration of mechanical ventilation, time to 2019 nCoV RT-PCR negativity) and one outcome for critical type (all-cause mortality).

We identified a total of 8,094 studies, and 5,267 remained after duplicates were removed. After screening the titles or abstracts of 5,267 publications, 2,011 were excluded, 3,256 articles were screened for eligibility, and 47 articles underwent detailed review. Seven articles were included in the final meta-analysis for intubation outcome, six articles were included in the correlation of LUS score to PO_2_/FiO_2_ outcome, and six articles were included in the qualitative synthesis for mortality outcome (Figure 1). Bosso^36^ and Rojatti^37^ papers are both included in the mortality and correlation to PO_2_/FiO_2_ outcomes, and de Alencar^38^ is included in both intubation and mortality outcomes.

We extracted information from 16 articles according to predefined criteria. The included studies used LUS in PCR-confirmed COVID-19-positive patients and had been published between March 2020–November 2021 with sample sizes ranging from 10 in Dargent 2020^39^ and Tan 2020^40^ to 312 in Secco 2021.^41^ Retrospective studies predominated. There was significant heterogeneity between the studies regarding ultrasound protocols, performing personnel, and outcomes reported.

For the meta-analysis, 11 prospective studies, five retrospective studies, and one cross-sectional study were identified (Table 1). The studies in the meta-analysis were all conducted outside the United States, namely in Brazil, France, China, Italy, Sweden, and Israel. Between the initial time frame of search and data analysis, Lu et al^42^ had been retracted, and so we did not include it. We regarded the published data as sufficient to perform meta-analysis on LUS score correlation to intubation rates and PO_2_/FiO_2_ and quantitative synthesis on mortality outcome. Other reviewed studies were excluded due to population, age, use of different scoring systems, non-English language of publication, and case studies (Supplement 2).

**Table 1.** Overview of study characteristics of included studies.

	Design	N	Setting	LUS scoring	US operators	Outcomes
Bonadia 2020^53^	Single-center prospective cohort	41	ED	14 zones	ED staff 5 years POCUS experience	Mortality, LUS patterns correlation with ICU and invasive ventilation
Bosso 2020^36^	Single-center prospective observational	53	COVID-19 unit	12 zones	Expert clinicians	Mortality, degree of hypoxemia
Castelao 2021^45^	Single-center prospective observational	63	Inpatient and respiratory intermediate care unit	12 zones	Unknown operator	Distribution of US findings, LUS correlation with P/F ratio
Dargent 2020^39^	Single-center prospective observational	10	ICU patients	12 zones	LUS trained practitioners until interobserver agreement	Clinical course, intubation, ventilator associated pneumonia
De Alencar 2021^38^	Single-center prospective cohort	180	ED	12 zones	Emergency physicians	Death, intubation, ICU admission
Deng 2020^20^	Single-center retrospective cohort	128	ICU patients	8 zones WINFOCUS	Sonographers with 2–10 years experience blinded and undefended observers	Correlation of LUS scores to CT scores
Duclos 2021^46^	Multicenter retrospective observational	57	ICU	12 zones	LUS operators-academic teacher with publications or expert	LUS to predict 28-day mortality
Li 2021^48^	Single-center prospective observational cohort	48	ICU	12 zones	Unknown, then senior ICU physician CCUSG certified interpretation	LUS score correlation to PaO_2_/FiO_2_, APACHE II, 28-day mortality
Lichter 2020^49^	Single-center retrospective observational	120	ICU and inpatients	12 zones	3 cardiologists	All-cause mortality and composite endpoint composed of death or new need for invasive mechanical ventilation
Perrone 2021^54^	Single-center prospective cohort	52	Internal medicine ward	14 zones	Expert physician >15 years of experience in thoracic US	LUS score association to clinical worsening- high flow oxygen support, ICU admission, or 30-day mortality
Persona 2021^47^	Single-center prospective observational	28	ICU	12 zones	Unknown	LUS score in patients on admission and discharge from ICU
Rojatti 2020^37^	Two-center retrospective observational	41	ICU	8 zones	Unknown	Severity of gas exchange impairment and IL-6
Secco 2021^41^	Single-center prospective cohort	312	ED	12 zones	Emergency physicians	LUS score and mortality at 30 days
Seiler 2021^51^	Single-center prospective cohort	72	ICU and inpatients	12 zones	5 consultant anesthesiologists	LUS score and indication for invasive mechanical ventilation, PO_2_/FiO_2_
Sumbul 2021^52^	Single-center cross-sectional	44	ICU and inpatient	12 zones	Two radiology specialists experienced in lung US	Modified LUS and severity of disease, PO_2_/FiO_2_ and pro-BNP
Tan 2020^40^	Single-center prospective cohort	12	ICU or isolation ward	10 zones; Buda scoring system for interstitial lung disease	ICU physicians received training and obtained qualifications	Modified LUS to evaluate the severity and treatment of COVID-19
Zieleskiewicz 2020^16^	Multicenter retrospective observational	100	ED and ICU	12 zones	Emergency or ICU physicians	LUS vs chest CT for assessment of COVID-19 pneumonia

*LUS*, lung ultrasound; *US*, ultrasound; *POCUS*, point-of-care ultrasound; *ED*, emergency department; *ICU*, intensive care unit, *CT*, computed tomography; COVID-19, coronavirus 2019; *IL-6*, interleukin-6; *PO_2_/FiO_2_*, ratio of partial pressure of oxygen in arterial blood to the fraction of inspiratory oxygen concentration; *BNP*, B-type natriuretic peptide.

There was significant heterogeneity between studies regarding ultrasound protocols. The LUS protocols systematically evaluate lung parenchyma by the examination of anatomic zones of each thorax. Each hemithorax is systematically divided into regions for evaluation: two anterior, two lateral, and two posterior demarcated by anatomical landmarks set by the anterior and posterior axillary lines. Each region is then divided into superior and inferior halves for ultrasonographic examination. In each zone, findings of a normal lung pattern receive a score of 0; well defined B lines receive a score of 1; coalescent B lines are scored as 2; and findings of parenchymal consolidation are scored as 3. The sum of scores assigned to each lung field on both hemithoraces is tabulated and comprises the LUS score.

An 8-zone protocol, described by Volpicelli,^43^ was used by Deng^20^ and Rojatti^37^ and evaluated two anterior and two lateral zones per hemithorax. The posterior lung fields are omitted from evaluation in the 8-zone Volpicelli protocol and are subsequently included in protocols with additional views. The 10-zone protocol used by Tan^40^ evaluates one additional posterior lung field on each hemithorax compared to the 8-zone Volpicelli protocol. The 12-zone evaluation, commonly used in the BLUE protocol^44^ evaluates two additional lung fields. In addition to the anterior and lateral locations, this protocol includes one inferior and one superior zone. The 12-zone protocol was used by Bosso,^36^ Castelao,^45^ Dargent,^39^ Duclos,^46^ de Alencar,^38^ Persona,^47^ Li,^48^ Lichter,^49^ Secco,^50^ Seiler,^51^ Sumbul,^52^ and Zieleskiewicz^16^ studies. Lastly, the 14-zone protocol used by Bonadia^53^ and Perrone^54^ was described by Soldati et al^21^ in 2020. The protocol evaluates an additional three posterior lung fields on each hemithorax in addition to the two anterior and lateral locations. All study protocols used curvilinear probes except for Lichter,^49^ which used a phased array probe for evaluation.

Ultrasounds were performed by a range of personnel from cardiologists and sonographers to ED and ICU staff with varying levels of training and experience. All the analyzed studies but Rojatti described the experience of the ultrasound operators. No training protocol assessments were discussed, except for Dargent, which trained operators until good inter-observer reliability was achieved. Interpretations of images were also performed by personnel with differing levels of training ranging from study authors to radiologists to cardiologists. Since ultrasound is heavily operator-dependent this may have contributed to the heterogeneity of results.

The QUADAS-2 review (Supplement 3) showed that most studies had significant patient selection biases. Some studies enrolled convenience samples rather than consecutive patients due to resource constraints. Studies excluded patients with history of congestive heart failure, interstitial lung disease, pneumothorax, patients who were unable to sit up or participate in an exam, or who had DNR/DNI status, <6-month life expectancy, congenital heart disease, or recent chest surgery. While these exclusions may have affected accuracy of outcome results given that the presence of comorbidities increases morbidity and mortality, it also served to make the LUS findings more specific for COVID-19.

## RESULTS

**Figure 2.**
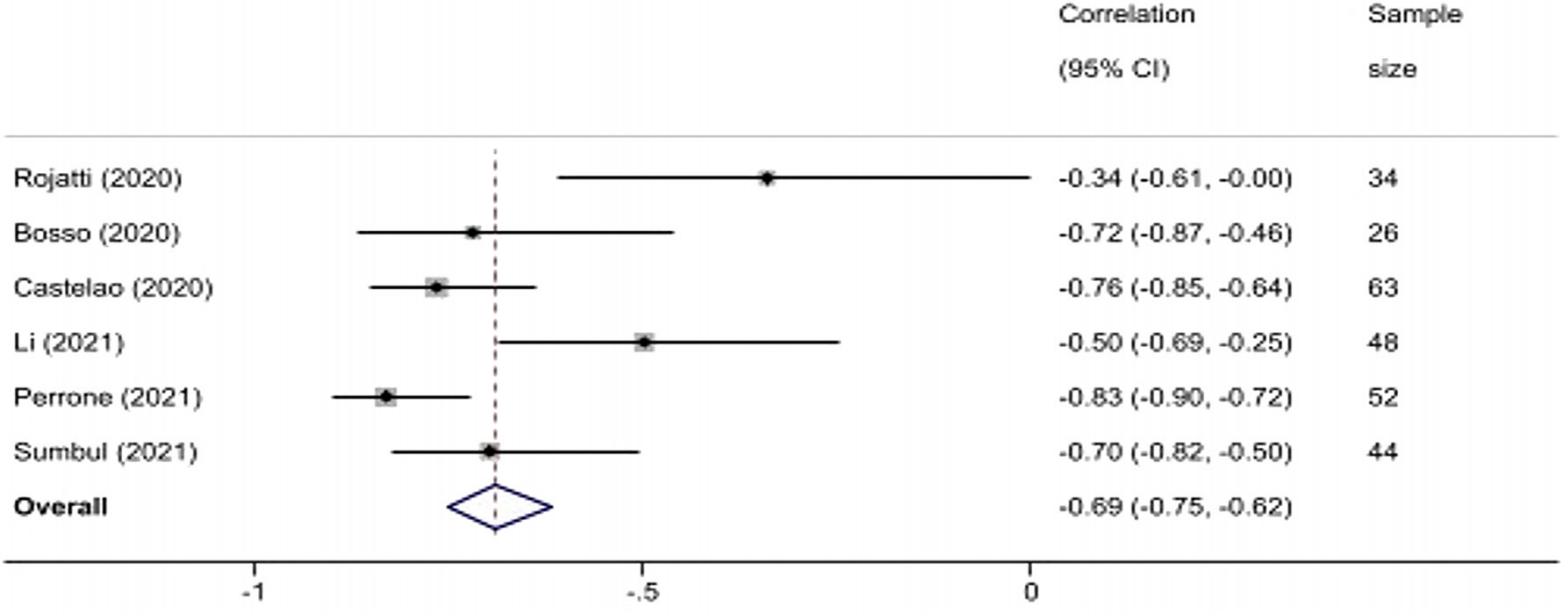
Forest plot of correlation between lung ultrasound and PO_2_/FiO_2_. In pooled results, the correlation coefficient was −0.69 (95% −0.75, −0.62). There was significant heterogeneity between the studies as measured by the I^2^ and Cochran Q test. *CI*, confidence interval; *PO*_*2*_ */FiO*_*2*_, ratio of partial pressure of oxygen in arterial blood to the fraction of inspiratory oxygen concentration.

In the six studies included in the meta-analysis focused on the correlation between LUS score and PO_2_/FiO_2_, there were a total of 267 patients. We found a significant negative correlation between increasing LUS score and pulmonary gas exchange measurement of PO_2_/FiO_2._ In pooled results, the correlation coefficient was −0.69 (95% −0.75, −0.62). There was significant heterogeneity between the studies as measured by the I^2^ and Cochran Q test. Rojatti^37^ and Li^48^ studies included only patients in the ICU while other studies were performed on patients in COVID-19 units (Bosso,^36^ Castelao,^45^ Sumbul,^2^) and hospital ward (Perrone^54^). See Figure 2.

**Figure 3.**
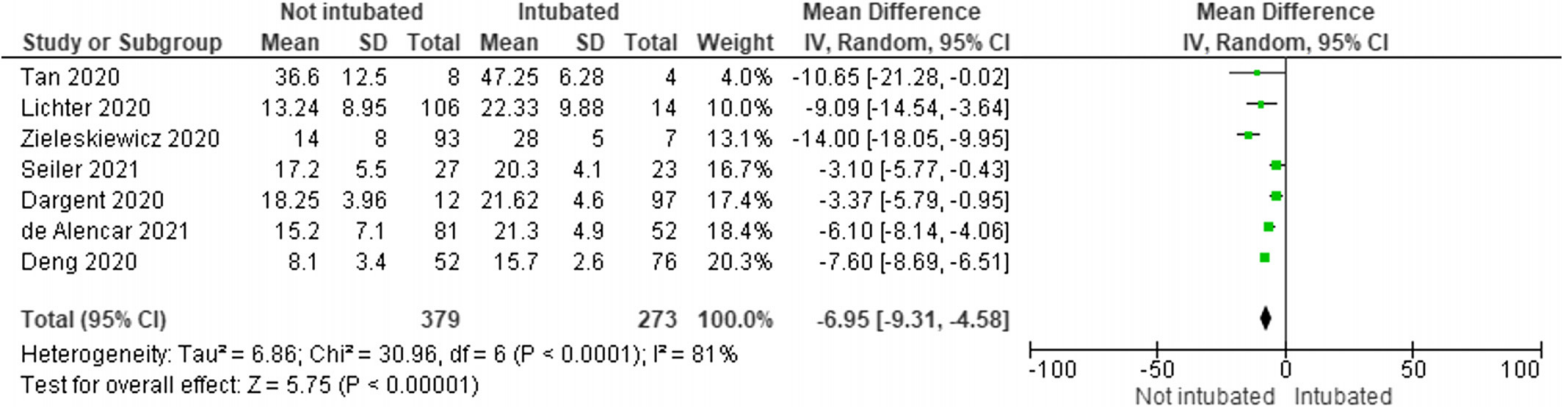
Differences in lung ultrasound (LUS) scores for intubated/non-intubated subjects. I^2^ of 81% and Cochran Q test show significant heterogeneity between the studies of LUS scores of intubated vs non-intubated patients.

The meta-analysis comparing LUS scores for the intubation outcome included 273 intubated and 379 non-intubated patients. In pooled results, intubated patients had a mean LUS score that was 6.95 points higher (95% CI 4.58–9.31) than that of non-intubated patients. Mean LUS scores for intubated patients ranged from 15.7 (SD 2.6) in Deng 2020 to 47.25 (SD 6.28) in Tan 2020. The mean LUS score of the remaining studies fell between these values. Mean LUS scores for non-intubated patients ranged from 8.1 (SD 3.4) in Deng 2020 up to 36.6 (SD 12.5) in Tan 2020. Notably, Deng^20^ used an 8-zone LUS score while Tan^40^ used a 10-zone LUS score, which may partially account for the large spread of LUS score results (Figure 3).

**Figure 4.**
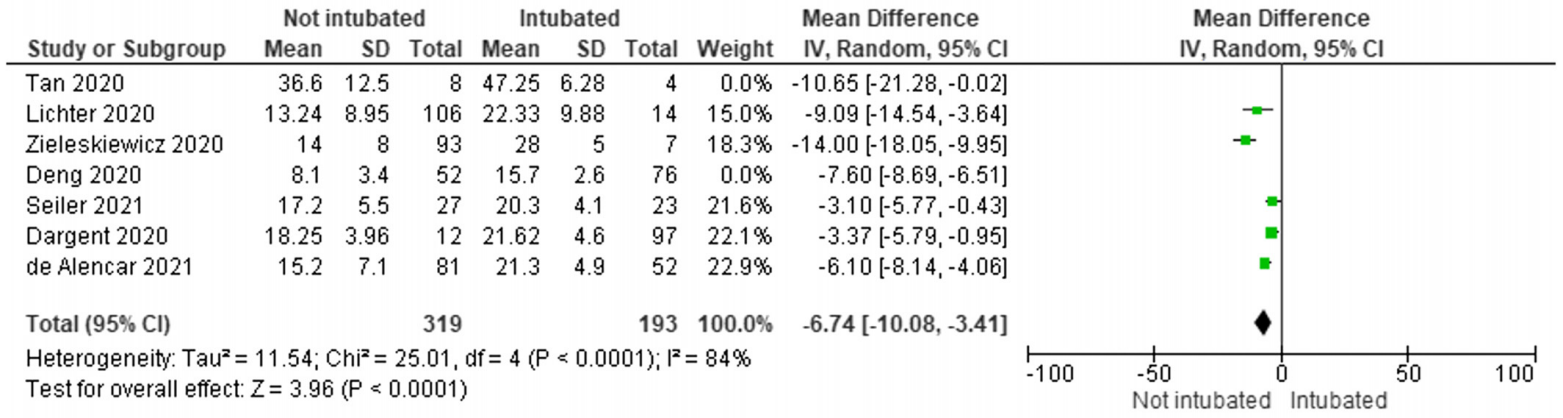
Differences in lung ultrasound (LUS) scores for intubated/non-intubated subjects in subgroup analysis of 12-zone protocol studies. I^2^ of 84% and Cochran Q test show significant heterogeneity between the studies of LUS scores of intubated vs non-intubated patients.

Subgroup analysis was performed on the studies that used the 12-zone protocol (Lichter,^49^ Zieleskiewicz,^16^ Seiler,^51^ Dargent,^39^ de Alencar^38^) as the most frequently used protocol. In pooled results of the subgroup analysis, the 193 intubated patients had a mean LUS score that was 6.74 points higher (95% CI 3.41–10.08) than that of the 319 non-intubated patients (Figure 4). Protocol notwithstanding, LUS scores were higher in intubated patients than non-intubated patients consistent with the finding that LUS score increases with more diffuse lung involvement^19^ and, therefore, severity of illness. There was significant heterogeneity between the studies as measured by the I^2^ and Cochran Q test.

**Figure 5.**
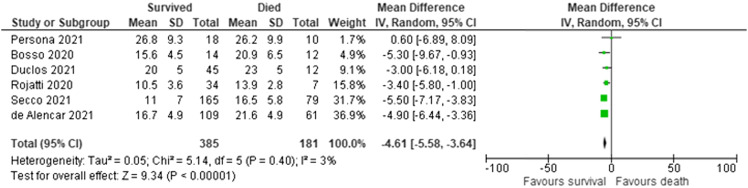
Differences in lung ultrasound (LUS) scores for survivors vs non-survivors. The I^2^ of 3% and the p-value for heterogeneity of 0.4 show little evidence of publication bias in the included studies of LUS scores for survivors vs non-survivors.

In the six studies included in the quantitative analysis of mortality, there was a total of 566 patients, with 385 patients who survived and 181 who did not survive. In pooled results, survivors had a mean LUS score that was 4.61 points lower (95% CI 3.64–5.5) than that of non-survivors. The LUS scores of those who survived ranged from 11 (SD 7) in Secco 2021^41^ up to 26.8 (SD 9.3) in Persona 2021.^47^ The LUS scores of non-survivors ranged from 13.9 (SD 2.8) in Rojatti 2020^37^ up to 26.2 (SD 9.9 in Persona 2021.^47^ Secco 2021 was conducted in an ED setting while Persona 2021^47^ and Rojatti 2020^37^ used patients in an ICU setting. Depending on the patient population and factors in the study location epidemiology, ED settings may have had a patient population less critically ill than patients in ICU, which would have led to the studies conducted in EDs to have baseline lower LUS scores. A study using a 12-zone protocol also contributes to higher overall LUS scores since LUS score is calculated with the cumulative scores of the number of zones. Persona^47^ and Secco^41^ used the 12-zone protocol, while Rojatti 2020^37^ used the 8-zone protocol (Figure 5).

**Figure 6.**
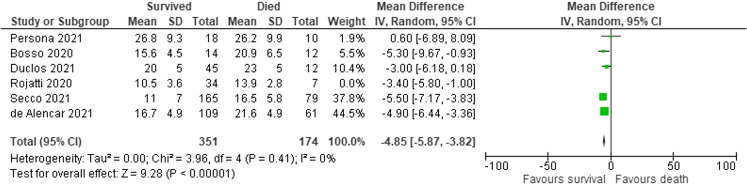
Differences in lung ultrasound (LUS) scores for survivors vs non-survivors in subgroup analysis of 12-zone protocol studies. The I^2^ of 0% and the *P*-value for heterogeneity of 0.4 show little evidence of publication bias in the included studies of LUS scores for survivors vs non-survivors.

Subgroup analysis was performed on the studies using the 12-zone protocol (Persona,^47^ Bosso,^36^ Duclos,^46^ Secco,^41^ de Alencar^38^). In pooled results of the subgroup analysis, the 351 survivors had a mean LUS score that was 4.85 points lower (95% CI 3.82–5.87) than that of the 174 non-survivors (Figure 6). Despite the different ultrasound protocols, patients with lower ultrasound score and, therefore, less lung involvement^19^ were found to be more likely to survive. Among the included studies, there was no evidence of significant heterogeneity, as measured by the I^2^ and Cochran Q test.

Mean scores did appear higher in intubated patients than in patients who died. We speculate that this could have been multifactorial, possibly secondary to timing of scans in disease course and limitations of resource allocation in height of the pandemic. The de Alencar^38^ study, which looked at both intubation and mortality outcomes, had a LUS score that would be as expected—higher in intubated patients and higher still in patients who died.

## DISCUSSION

Our meta-analysis found that a higher LUS score was strongly correlated with a decreasing PO_2_/FiO_2_ in patients diagnosed with COVID-19 pneumonia. The LUS score was also found to be significantly higher in intubated vs non-intubated patients and in critically ill patients who did not survive with COVID-19 pneumonia.

The LUS has been well established in the diagnosis of pneumothorax, lung consolidation, alveolar-interstitial syndrome and pleural effusion.^55^ We sought to determine whether LUS abnormalities in COVID-19 patients correlated to abnormalities in pulmonary gas exchange as a LUS score was found to be a valid tool to assess regional and global lung aeration.^56^ Our quantitative meta-analysis found that LUS score was inversely correlated to PaO_2_/FiO_2_ ratio, which would be expected. As LUS score increases in COVID-19 with increasing interstitial edema and consolidation, lung aeration worsens, thereby causing an increase in shunting and hypoxemia and a decrease in the PaO_2_/FiO_2_ ratio. The correlation of an increasing LUS with worsening PaO_2_/FiO_2_ ratio and increasing intubation rates suggests that ultrasonographic monitoring reflects illness severity and disease progression. This indicates the potential value of LUS for dynamic lung monitoring as reported by Deng,^20^ Dargent^39^ in the ICU population, and Casella^57^ in the non-ICU setting. Patients with COVID-19 at higher risk of adverse outcomes may benefit from more intensive monitoring or earlier intervention with noninvasive respiratory support in anticipation of deteriorating clinical course.

In pooled results, we found significant correlation between LUS score and mortality rates in patients with COVID-19 pneumonia. Various published studies have looked at LUS cutoffs for mortality and adverse outcomes. Ji found LUS score >12 predicted adverse outcomes with a specificity and sensitivity of 90.5% and 91.9%,^59^ while Secco found LUS score >13 had a 77.2% sensitivity and a 71.5% specificity in predicting mortality.^50^ Sun found that LUS score >15 had a sensitivity of 92.9% and specificity of 85.3% for prediction of mortality,^60^ while Lichter found that mortality increased with LUS score >18.^49^ De Alencar found LUS score ≥26 had 90% specificity for mortality,^38^ and Li found that for LUS score >22.5, the sensitivity and specificity were 83.3% and 72.2% for predicting mortality.^48^ Finally, Trias-Sabra found that LUS score ≥24 had a higher risk of ICU admission or death.^61^ There is currently no consensus, which we speculate is secondary to the various ultrasound protocol used, since the number of zones measured has a direct effect on the cumulative LUS score.

We chose ultrasound protocols in an attemp to find the optimal balance between the acquisition time and accuracy. There is no standardized LUS protocol for the evaluation of COVID-19 pneumonia, with current protocols ranging from an 8-zone evaluation^43^ to a 14-zone evaluation^21^ with nominal scale. Protocols also often required modification in supine critically ill patients, as posterior segments were difficult to evaluate. Soldati^21^ proposed a 14-point protocol modified to 7 points in critically ill supine patients for the international standardization of the use of LUS in COVID-19.

A study comparing the different protocols showed that the posterior areas are fundamental to capture the most important findings in patients with COVID-19 pneumonia.^62^ A 12-zone system maintains balance between acquisition time and accuracy, although a 10-point system is sufficiently accurate if the basal posterior regions are included.^62^ Recently, an abbreviated 8-zone protocol was found to be as accurate as the previously validated 12-zone protocol for prognostication of clinical deterioration in non-ventilated COVID-19 patients. Scanning times were 50% shorter in the 8- vs 12-zone protocol, although specific times were not delineated. ^63^ A shorter protocol with sufficient accuracy could decrease risk of contagion by limiting operator exposure and thereby increase operator safety.

A LUS has been reported to have higher sensitivity than CXR, especially early in infection, for detecting COVID-19-associated lung lesions with a reported sensitive of 92–96% compared to 46–69% for CXR.^64^ ^–^ ^68^ Lichter^49^ found that higher LUS score predicted intubation and mortality independent of CXR findings. Patients with a higher percentage of lung involvement on CXR were found to have higher intubation rates^69^ ^,^ ^70^ ^,^ ^71^ ^,^ ^72^ as well as higher mortality.^69^ ^,^ ^73^ Spogis^74^ found that changes in CXR appeared more sensitive for predicting ICU treatment than LUS; however, LUS was more specific. Both modalities were found to be good discriminators with each modality having its own advantages and disadvantages.

Advantages of CXR include its wide availability, lack of examiner dependency, ease of comparing previous examinations, and ability to examine the entire lung in one image. A LUS can produce real-time dynamic images and is accurate, reproducible, without ionizing radiation, and easily disinfected. However, LUS requires more time to perform than CXR increasing exposure risk to clinician. There may be greater total time from CXR performance to interpretation depending on the individuals who are performing and interpreting the scans. Advantages of one modality over another may be institutional, resource, and patient dependent.

The results of this meta-analysis and systematic review show that the LUS score has significant correlation to PO_2_/FiO_2_ ratio and to clinical outcomes of intubation rate and mortality in COVID-19 positive patients with pneumonia. Especially in cases of surge capacity, this would provide important prognostication information to aid clinicians in resource allocation and the identification of patients at a higher risk of deterioration for the appropriate level of care. The LUS score contributes to the classification of disease severity and the monitoring of disease progression, and it can influence the decision to escalate drug treatment or early ventilatory support. It also has the advantage of reducing the number of exposed healthcare workers, limiting resource consumption and environmental contamination. Implementation of bedside LUS will be dictated by specific institutional workflows, resource availability, and patient volume. Timely and accurate classification of patients is crucial during the pandemic since the excessive influx of patients can place hospital and patient care organizations in crisis and alter the efficiency and services of EDs.

## LIMITATIONS

Limitations of POCUS LUS include the inability to evaluate lung lesions that are deep and intrapulmonary, difficulty in scanning posterior basilar regions, and relative lower sensitivity than CT. A LUS has lower specificity than CT for COVID-19 as B lines can also be found in pulmonary edema due to cardiac disease, pulmonary aspiration, ARDS, interstitial lung disease, or pneumonia.^43^ Subpleural consolidations and effusions are observed in both COVID-19 and other viral and non-viral pneumonia and pulmonary embolism.^43^ A LUS needs to be used in conjunction with other confirmatory tests such as PCR for increased accuracy.

There was significant selection bias in included studies. Studies did not include COVID-19 patients with symptoms that were extra-pulmonary in nature, which currently include gastrointestinal symptoms, anosmia, ageusia, rhinorrhea, and altered mental status.^17^ It is unclear whether patients with other presenting symptoms would have an abnormal LUS, which would make LUS less sensitive as a testing modality. In addition, many studies did not exclude patients with baseline pulmonary disease and comorbidities that may alter baseline LUS. A LUS was often performed in patients with worse illness severity, also contributing to selection bias.

Additional limitations of this meta-analysis include study heterogeneity, lack of a standardized guideline for POCUS lung evaluation in COVID-19, performance of LUS by operators with different levels of training, and a lack of specified training protocol. Lack of unifying definitions and inconsistencies with reporting COVID-19 lung abnormalities limit comparisons between different studies, geographical areas, and patients.

## CONCLUSION

This meta-analysis shows that a higher lung ultrasound score is significantly negatively correlated to PaO_2_/FiO_2_ and positively correlated to intubation rates and mortality rates in COVID-19 positive patients with pneumonia. In the ED and ICU settings, a LUS score may be a useful modality in determining patient disposition and aiding in prognostication of care and resource allocation.

## Supplementary Information







