# Supplementary material for: Lung Ultrasound Score in COVID-19 Patients Correlates with PO2/FiO2, Intubation Rates, and Mortality
Source: West J Emerg Med. 2023 Dec 22;25(1):28–39. doi: 10.5811/westjem.59975 (PMC10777190; doi:10.5811/westjem.59975)
Supplement: Supplementary file 1 [file wjem-25-28-s001.docx]

**Appendix 1**

**Search strategy**

(("wuhan"[tw] AND ("Coronavirus"[tw] OR "corona virus"[tw])) OR "nCov"[tw] OR "2019 ncov"[tw] OR "novel coronavirus"[tw] OR "novel corona virus"[tw] OR "covid-19"[tw] OR "SARS-COV-2"[tw] OR "Severe Acute Respiratory Syndrome Coronavirus 2"[tw] OR "coronavirus disease 2019"[tw] OR "corona virus disease 2019"[tw] OR "new coronavirus"[tw] OR "new corona virus"[tw] OR "new coronaviruses"[All Fields] OR "novel coronaviruses"[All Fields] OR "Severe Acute Respiratory Syndrome Coronavirus 2"[Supplementary Concept] OR "2019 ncov"[tw] OR "ncov 2019"[tw] OR "sars coronavirus 2"[All Fields]) AND ("ultrasonography/methods*"[mesh] OR "ultrasound"[tiab] OR "POCUS"[tiab] OR "point of care ultrasound"[tiab] OR "lung ultrasound"[tiab] OR "lung ultrasound score"[tiab] OR "LUSS"[tiab])
