# Supplementary material for: Lung Ultrasound Score in COVID-19 Patients Correlates with PO2/FiO2, Intubation Rates, and Mortality
Source: West J Emerg Med. 2023 Dec 22;25(1):28–39. doi: 10.5811/westjem.59975 (PMC10777190; doi:10.5811/westjem.59975)
Supplement: Supplementary file 2 [file wjem-25-28-s002.docx]

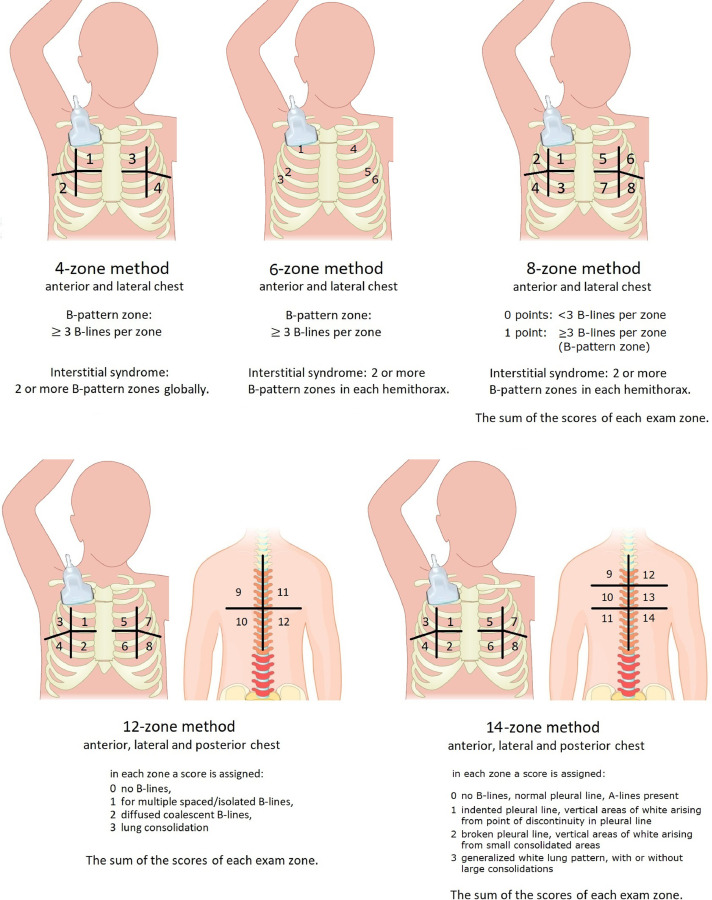


Supplement 1. Excerpt from Allinovi M, Parise A, Giacalone M, et al. Lung Ultrasound May Support Diagnosis and Monitoring of COVID-19 Pneumonia. *Ultrasound Med Biol*. Nov 2020;46(11):2908-2917.
