# Supplementary material for: Lung Ultrasound Score in COVID-19 Patients Correlates with PO2/FiO2, Intubation Rates, and Mortality
Source: West J Emerg Med. 2023 Dec 22;25(1):28–39. doi: 10.5811/westjem.59975 (PMC10777190; doi:10.5811/westjem.59975)
Supplement: Supplementary file 3 [file wjem-25-28-s003.docx]

Supplement 3. Bias assessment using the QUADAS-2 tool

| **Study** | **RISK OF BIAS** | | | | | | **APPLICABILITY CONCERNS** | | |
| --- | --- | --- | --- | --- | --- | --- | --- | --- | --- |
|  | | **PATIENT SELECTION** | | **INDEX TEST** | **REFERENCE STANDARD** | **FLOW AND TIMING** | **PATIENT SELECTION** | **INDEX TEST** | **REFERENCE STANDARD** |
| Bonadia 2020 | ☹ | | | ☹ | n/a | ? | ☹ | ? | n/a |
| Bosso 2020 | ? | | | ☹ | n/a | ? | ? | ☹ | n/a |
| Castelao 2020 | ? | | | ☹ | ? | ☺ | ☺ | ☺ | ☺ |
| Dargent 2020 | ? | | | ? | ☹ | ☹ | ☺ | ☹ | ☹ |
| De Alencar 2021 | ☹ | | | ☹ | n/a | ☹ | ☹ | ☹ | n/a |
| Deng 2020 | ☹ | | | ☺ | ☺ | ☹ | ☺ | ☺ | ☹ |
| Duclos 2021 | ☹ | | | ☺ | ? | ☹ | ☺ | ☺ | ☹ |
| Li 2021 | ☺ | | | ? | n/a | ☺ | ☺ | ☺ | n/a |
| Lichter 2020 | ☹ | | | ☺ | n/a | ☹ | ☺ | ☺ | n/a |
| Perrone 2021 | ☹ | | | ? | n/a | ? | ☺ | ☺ | n/a |
| Persona 2021 | ☺ | | | ? | n/a | ☺ | ☺ | ☺ | n/a |
| Rojatti 2020 | ? | | | ? | n/a | ☹ | ☹ | ? | n/a |
| Secco 2021 | ☺ | | | ☺ | n/a | ☺ | ☺ | ☺ | n/a |
| Seiler 2021 | ☹ | | | ☹ | n/a | ☺ | ☹ | ☺ | n/a |
| Sumbul 2021 | ☹ | | | ☹ | n/a | ? | ☺ | ☺ | n/a |
| Tan 2020 | ☹ | | | ? | ? | ? | ☹ | ☹ | ☹ |
| Zieleskiewicz 2020 | | | ☹ | ☹ | ☺ | ☺ | ☹ | ☹ | ☺ |

☺Low Risk ☹High Risk ? Unclear Risk
